# Effects of Grinding Methods of Tartary Buckwheat Leaf Powder on the Characteristics and Micromorphology of Wheat Dough

**DOI:** 10.3390/foods13081233

**Published:** 2024-04-17

**Authors:** Yuxia Feng, Jiaying Zhu, Yunlong Li, Zhe Cheng

**Affiliations:** Institute of Functional Food of Shanxi, Shanxi Agricultural University, Taiyuan 030031, China; z20213495@stu.sxau.edu.cn (Y.F.); z20223599@stu.sxau.edu.cn (J.Z.); chengzhe@sxau.edu.cn (Z.C.)

**Keywords:** tartary buckwheat leaf powder, dough, hydration characteristics, rheological properties, microstructure

## Abstract

The functional components in tartary buckwheat leaf powder can give flour products higher nutritional value. To comprehensively realize the high-value utilization of tartary buckwheat and its by-products, electric stone mill powder (EMP), ultra-fine mill powder (UMP), steel mill powder (SMP), and grain mill powder (GMP) from tartary buckwheat leaves were used in the preparation of wheat dough, and this was used to explore their effects on dough properties and protein microstructure. With an increase in tartary buckwheat leaf powder, the hydration characteristics, protein weakening rate, and starch gelatinization characteristics of the dough changed, and the water holding capacity and swelling capacity decreased. The retrogradation value increased, which could prolong the shelf life of related products. The water solubility of the dough showed an upward trend and was the lowest at 10% UMP. The addition of UMP produced a more uniform dough stability time and the lowest degree of protein weakening, which made the dough more resistant to kneading. An increasing amount of tartary buckwheat leaf powder augmented the free sulfhydryl content of the dough and decreased the disulfide bond content. The disulfide bond content of the dough containing UMP was higher than that of the other doughs, and the stability of the dough was better. The peaks of the infrared spectrum of the dough changed after adding 10% UMP and 20% EMP. The content of α-helical structures was the highest at 10% UMP, and the content of ordered structures was enhanced. The polymerization of low molecular weight proteins to form macromolecular polymers led to a reduction in surface hydrophobic regions and the aggregation of hydrophobic groups. The SEM results also demonstrated that at 10% tartary buckwheat leaf powder, the addition of UMP was significantly different from that of the other three leaf powders, and at 20%, the addition of EMP substantially altered the structure of the dough proteins. Considering the effects of different milling methods and different added amounts of tartary buckwheat leaf powder on various characteristics of dough, 10% UMP is the most suitable amount to add to the dough.

## 1. Introduction

Tartary buckwheat has high nutritional value, containing protein, bioflavonoids, multiple vitamins, cellulose, 18 different amino acids, chlorophyll, magnesium, potassium, calcium, iron, manganese, zinc, chromium, copper, selenium, and other ingredients [1]. Tartary buckwheat leaves are rich in macroelements such as calcium, magnesium, and phosphorus, as well as essential trace elements such as iron, manganese, potassium, and tin, and they also contain a large number of flavonoids such as rutin, which have physiological activities and can effectively prevent cardiovascular diseases and capillary embrittlement [2,3].

At present, the research on tartary buckwheat has mainly focused on its seeds. Researchers have also studied the polyphenols in tartary buckwheat flowers, leaves, stems, and roots and found the highest rutin content in leaves [4]. Although tartary buckwheat leaves contain a large number of functional components such as rutin, carotenoids, lutein [5], and d-inositol [6], all of which have high nutritional and health-promoting value, the use of the leaves is still infrequent and underdeveloped. Tartary buckwheat cake made using tartary buckwheat leaf powder as the raw material was found to have an antioxidant capacity 2.27–2.99 times higher than that of wheat cake [7]. Researchers added bitter wheat leaves, hawthorn leaves, and ginkgo leaves to flour to make bread and peach crisp and found that adding three percent plant leaf powder to flour had no adverse effect on the sensory quality of the baked goods, and the loss of flavonoids in processing and baking was small [8]. Other researchers have developed a detoxifying health meal replacement powder [9]. However, the utilization of tartary buckwheat leaf powder is still infrequent, and there are fewer foods developed with tartary buckwheat leaves than with other raw materials.

The reason that tartary buckwheat leaves are not used often in food could be that they are not well known to the public. Adding them to noodles could not only improve the nutritional value of the noodles, but also expand the applications of tartary buckwheat leaves in food. Different grinding methods can affect the physical and chemical properties and pasting properties of flour, resulting in different degrees of impact on the processed products. The grinding treatment of the sample can not only improve the utilization of raw materials, but it is also a necessary process for the production of products. Different grinding methods will have different degrees of influence on the physical and chemical properties of grain powder. Different grinding methods will change due to the speed and heat production, and the powder processing characteristics of the grain powder will be affected by the mechanical strength. This study selected four types of commonly used grinding equipment to grind tartary buckwheat leaf powder. From there, we will enrich tartary buckwheat leaf products and introduce them into staple foods. In this study, tartary buckwheat leaf powder prepared by different milling methods was added to wheat flour to improve the nutritional value of wheat flour. The addition of tartary buckwheat leaf powder will change the network structure of dough. By analyzing the hydration characteristics, rheological properties, internal disulfide bond content, and protein structure of the dough, the effects of adding tartary buckwheat leaf powder on the processing characteristics and microstructure of wheat dough were explored. The aim was to provide data and a theoretical basis for tartary buckwheat leaf powder noodles.

## 2. Materials and Equipment

### 2.1. Materials

Tartary buckwheat leaves were obtained from the Shanxi Agricultural University Functional Food Research Institute (Taiyuan, China). Wheat flour (protein: 12.20%, fat: 1.60%, starch: 73.00%, ash: 0.70%) was obtained from Wudeli Food Flour Co., Ltd. (Handan, China). Rutin, γ-aminobutyric acid (GABA), and gallic acid were purchased from Yuanye Biology Co., Ltd. (Shanghai, China). All chemicals used were of analytical grade.

### 2.2. Preparation of Mixed Powder, Dough, and Protein

#### 2.2.1. Preparation of Mixed Powder

The tartary buckwheat leaves were dried at 60 °C, ground by four different milling methods to form: electric stone mill powder (EMP), (Bluestone 45 × 65, Quanzhou Grinding Edge Electric Stone Mill Co., Ltd., Quanzhou, China), where the rotation speed was 24 r/min; ultra-fine mill powder (UMP) (RT-UF26, Rongcong Precision Technology Co., Ltd., Taiwan, China), where the rotation speed was 25,000 r/min; steel mill powder (SMP), (LG30, Tianjin Taisite Instrument Co., Ltd., Tianjin, China), where the rotation speed was 24,000 r/min; and grain mill powder (GMP), (HK-860, Guangzhou Xulang Machinery Equipment Co., Ltd., Guangzhou, China), where the rotation speed was 1420 r/min. The tartary buckwheat leaves were separately fed into different devices for grinding, and the samples were taken out from the discharge port, until all the powder was passed through an 80-mesh sieve. Tartary buckwheat leaf powder prepared by different milling methods was added to wheat flour, and the proportion of buckwheat leaf powder was 0%, 10%, 20%, and 30%. The two powders were mixed evenly and stored at 4 °C.

#### 2.2.2. Preparation of Dough

One hundred grams of each flour mix (0%, 10%, 20%, or 30% tartary buckwheat leaf powder) was added to the mixer (Little Bear Electric Co., Ltd., Foshan, China) with 60 mL of distilled water and mixed until the dough was formed. The dough was placed in a dough press at a roll pitch of 5 mm and rolled 3–4 times until a dense dough sheet without holes was formed.

#### 2.2.3. Extraction of Dough Protein

A portion of the dough was subjected to alkali extraction and acid precipitation to obtain gluten protein. The dough and extracted protein were then lyophilized, ground through an 80-mesh sieve, and stored at 4 °C, which were used to determine its microstructure.

### 2.3. Determination of Particle Size of Tartary Buckwheat Leaf Powder

The powder was dispersed in ethanol at a ratio of 1:300 (*w*/*v*) and analyzed using a laser diffraction particle size analyzer (BT-2001, Dandong Baiter Instrument Co., Ltd., Dandong, China). The suspension was measured at a temperature of 20 ± 0.5 °C, with the laser obscuration range set at 10–20%. Each sample was measured at least three times. The cell rupture rate is a percentage that measures the degree of particle fragmentation or crushing, typically used to evaluate the effectiveness of the crushing process. The calculation formula is as follows.

Cell rupture% = $\left[1-\left(1-\frac{10}{D50}\right)\right]\times 100$; D50 is the median particle size, indicating the corresponding particle size when the cumulative particle size distribution percentage reaches 50%.

### 2.4. Determination of Dough Rheological Properties

The torque value of the kneaded dough after adding water was measured by two heating processes, and the rheological properties of the dough with different compositions were measured using a Mixolab 2 (automatic comprehensive powder analyzer; Chopin Technologies, Paris, France). The Chopin + 80 g program was used for assessment. During the experiment, if the target torque C1 value was not within 1.10 ± 0.05 N·m, the estimated water absorption rate was adjusted by altering the amount of buckwheat leaf powder and water until the desired torque range was obtained.

### 2.5. Determination of Hydration Properties

The water-holding capacity (WHC), water solubility (WS), and swelling capacity (SC) were determined according to methods outlined by Zhang et al. [10].

### 2.6. Free Sulfhydryl (-SH) Content

The content of free sulfhydryl groups in the samples was determined by the method of Liu et al. [11], with some modifications using Ellman’s reagent. The lyophilized sample (50 mg) was dissolved in 2 mL Tris-glycine buffer (pH 7.2), mixed with guanidine hydrochloride (4.7 g), diluted to 10 mL with Tris-glycine buffer, shaken at 37 °C for 1 h, and then centrifuged at 13,000× *g* for 10 min. One milliliter of the supernatant was mixed with 3 mL of Tris-glycine buffer and 0.1 mL of Ellman’s reagent (5,5′-dithiobis-(2-nitrobenzoic acid) (DTNB), 4 mg/mL). The reaction was carried out at room temperature for 20 min. The absorbance was measured at 412 nm using an ultraviolet spectrophotometer (A380, Shanghai, China).

### 2.7. Fourier Transform Infrared (FTIR) Spectroscopy Analysis

The samples and spectral-pure KBr (Kermal, SP, CAS: 7758-02-3) were dried at 40 °C for 8 h, mixed at a ratio of 1:100, and ground in an agate mortar before pressing into 20 mg thin disks at 10 lbs. The background was scanned 32 times from 400–4000 cm^−1^ at a resolution of 4 cm^−1^ (Nicolex is5, Thermo Scientific, Waltham, MA, USA). Omnic (version 8.0, Thermo Nicolet Inc., Waltham, MA, USA) and PeakFit (version 4.12, SPSS Inc., Chicago, IL, USA) were used to analyze the Fourier infrared spectra [12].

### 2.8. Fluorescence Spectrum Analysis 

The surface hydrophobicity of the gluten protein samples was determined using the 8-anilino-1-naphthalenesulfonic acid (ANS) fluorescent probe method as described in a previous study by Han et al. [13] with simple modifications. Briefly, 2 mg of a lyophilized sample was mixed with 1 mL of 50 mM acetic acid solution and shaken for 1 h at 37 °C. The supernatant was diluted to 1 mg/mL with an acetic acid solution. Twenty microliters of ANS (8.0 mM in the same buffer) was added to 4 mL of sample, and the fluorescence intensity was measured at 280 nm (excitation wavelength) and 410 nm (emission wavelength) using a Hitachi F4500 fluorescence spectrometer (Tokyo, Japan) with the slit width set at 5 nm.

### 2.9. Scanning Electron Microscope (SEM) Analysis 

Following the method reported by Sun et al. [14], lyophilized dough protein was selected for microstructure analysis. The samples were attached to the sample stage with conductive glue, sprayed with gold for 90 s, and cross-sections were observed with a cold-field SEM at 5.0 kV. Observations were made and photographed at magnifications of ×1000 and ×800.

### 2.10. Statistical Analysis

All tests were performed in triplicate. Two-way analysis of variance (ANOVA) was performed utilizing SPSS statistical software (19.0, SPSS Inc., Chicago, IL, USA). Differences in the means were determined by Duncan’s test at a significance level of 0.05.

## 3. Results and Discussion

### 3.1. Analysis of Properties of Tartary Buckwheat Leaf Powder

#### 3.1.1. Analysis of Basic Components of Tartary Buckwheat Leaf Powder

The basic components of the tartary buckwheat leaf powder were significantly different among the milling methods used (*p* < 0.05, Table 1). GMP had the highest ash content, followed by EMP, UMP, and SMP. The protein content of UMP was slightly higher than that of the other three milled powders, and there was no significant difference with GMP.

UMP had the highest content of the functional component GABA and SMP had the lowest. The content of rutin and quercetin was highest in UMP and lowest in EMP. The chlorophyll content was also significantly higher in UMP than in the other three milled powders. The results showed that the ultra-fine grinding method produced better and higher contents of functional factors such as rutin, quercetin, GABA, and chlorophyll.

The content of soluble dietary fiber was significantly higher in UMP than in the other milled powders (UMP > GMP > EMP > SMP), while the content of insoluble dietary fiber demonstrated the opposite trend. The total dietary fiber, which is the sum of soluble dietary fiber and insoluble dietary fiber, was 28.51 ± 0.08%, 28.53 ± 0.12%, 28.23 ± 0.09%, and 28.47 ± 0.03% for EMP, UMP, SMP, and GMP, respectively. UMP had a lower ratio of insoluble to soluble dietary fiber, which may be due to the high shear and friction of ultra-fine grinding that helps to grind the material into smaller particles. During this process, the cell walls of the tartary buckwheat leaves were destroyed, and the functional components in the cells were more easily extracted. In addition, ultra-fine grinding can change and refine the structure of proteins and fiber in the sample, promote the release of active ingredients, and increase the content of active ingredients in the tartary buckwheat leaf powder [15].

#### 3.1.2. Analysis of Particle Size Distribution and Specific Surface Area of Tartary Buckwheat Leaf Powder

The particle size of the powder is considered to be an important factor affecting the quality of grain flour, which to some extent reflects the magnitude of the mechanical strength exerted during grain grinding. The D50 values of buckwheat leaf powder prepared by four different grinding methods are shown in Table 2, arranged in descending order: GMP > SMP > UMP > EMP, with significant differences (*p* < 0.05). Among them, the particle size (D50) of leaf powder obtained from the GMP is 3–5 times larger than the other three methods, consistent with the significantly lower cell breakage rate compared to the other methods shown in Table 2. The grinding is carried out by the friction generated by the collision between the internal gears, resulting in relatively lower mechanical force among the four methods, which leads to insufficient grinding and a larger particle size of the buckwheat leaf powder. It is worth noting that the leaf powder processed by the electric stone mill method exhibits the smallest particle size (D50 = 2.59 ± 0.05) and the highest cell breakage rate. This may be related to the longer grinding time of this method compared to the other three methods and the fact that the stone mill itself is relatively heavy and the tartary buckwheat leaf is uniformly stressed in all parts. The combined effect of the larger grinding area, longer grinding time, and uniform stress results in a smaller D50 for buckwheat leaf powder. The finer the particle size of the leaf powder obtained by the four grinding methods, the larger the corresponding specific surface area.

**Figure 1:**
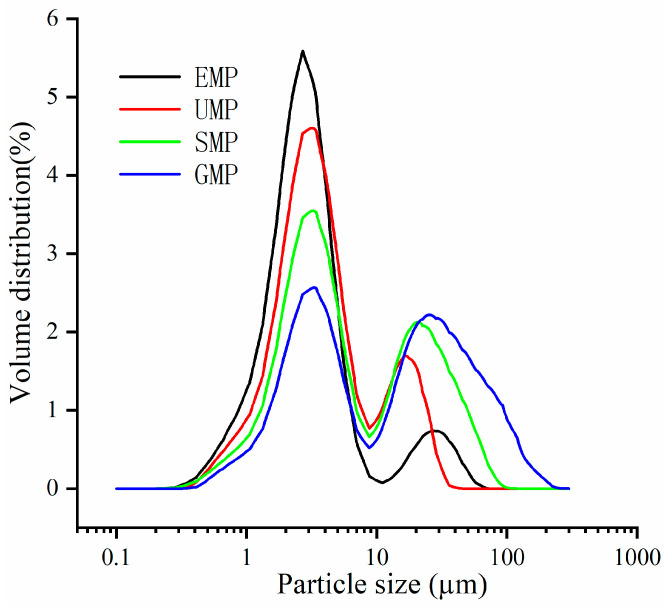
Particle size distribution curve of tartary buckwheat leaf powder under different milling methods.

Figure 1 shows the distribution of the four types of leaf powder, and each grinding method exhibits a bimodal distribution, meaning there are approximately two distinct particle size distributions. The difference between the small and large particle size distributions is not significant for GMP. As the D50 decreases, there is an increase in the distribution of small-particle-size leaf powder. In the region of large-particle-size distribution, the peak position of the ultra-fine grinding leaf powder is noticeably smaller than the other three methods.

#### 3.1.3. Analysis of Hydration Characteristics of Tartary Buckwheat Leaf Powder

The water holding capacity of GMP was significantly higher than that of the other three leaf powders under normal- and high-temperature conditions (Table 3). Studies have shown that a reduction in particle size can increase the surface area of the sample and increase the contact area with water, and ultra-fine grinding can change the spatial structure of the fiber material and increase the pores to easily combine with water [16,17]. However, the results of the present study were contradictory but consistent with those of Zhu et al. [18]. This may be due to the fact that the polysaccharide chains of insoluble dietary fiber in tartary buckwheat leaves were destroyed during the superfine grinding process, the hydrogen bonds of the polysaccharide chains were reduced, and the hydration ability was reduced. In addition, the water solubility of GMP was less than that of the other three leaf powders.

#### 3.1.4. Analysis of Infrared Spectrum Results of Tartary Buckwheat Leaf Powder

**Figure 2:**
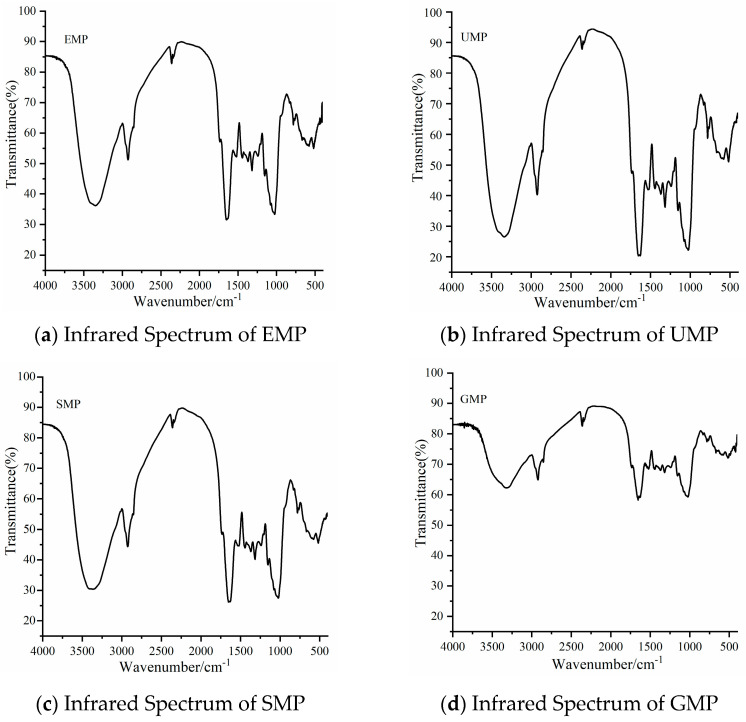
Infrared spectra of tartary buckwheat leaf powder obtained with different milling methods. (**a**) Infrared spectrum of EMP; (**b**) infrared spectrum of UMP; (**c**) infrared spectrum of SMP; (**d**) infrared spectrum of GMP.

As shown in Figure 2, the infrared spectrum peaks of tartary buckwheat leaf powder prepared by different grinding methods were roughly similar. The broad peak between 3000 and 3600 cm^−1^ is the stretching vibration peak of O–H in the structure of natural cellulose polysaccharides and polyphenols [19]. The absorption peak between 2800 and 3000 cm^−1^ is the stretching vibration of C–H on –CH_2_ or –CH_3_ in the hemicellulose polysaccharide [20]. The absorption peak at 1500–1650 cm^−1^ belongs to the stretching vibration of C=O and bending vibration of N–H, the characteristic absorption of amides [21,22]. The peak at 1156 cm^−1^ is caused by the tensile vibration of hemicellulose and cellulose, C–O–C. The broad absorption peak at 1022 cm^−1^ is the characteristic absorption peak of C–O–C in the hemicellulose sugar ring [23]. These peaks are characteristic of protein, cellulose, hemicellulose, and phenolic compounds, indicating that the four grinding methods did not destroy the basic structure of the components in tartary buckwheat leaf powder. However, the intensity of the above common characteristic peaks was significantly lower for GMP than for the other three powders.

#### 3.1.5. Effect of Tartary Buckwheat Leaf Powder on Dough Rheological Properties

**Figure 3:**
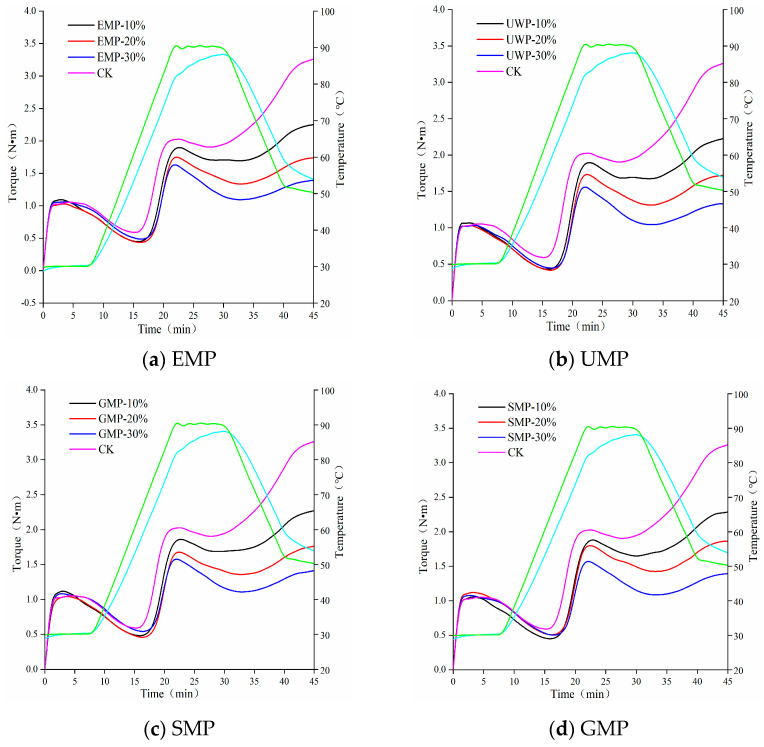
Mixolab curves of dough with different added amounts of tartary buckwheat leaf powder. (**a**) Mixolab curves of dough with different added amounts of EMP; (**b**) Mixolab curves of dough with different added amounts of UMP; (**c**) Mixolab curves of dough with different added amounts of SMP; (**d**) Mixolab curves of dough with different added amounts of GMP. CK indicates that the addition amount is 0%. All the blue curves in the figure represent the dough temperature, and the green curve represents the heating module temperature.

The heat flow characteristics of wheat flour mixed with different amounts of tartary buckwheat leaf powder obtained with the four different milling methods are shown in Figure 3. Rheological parameters are shown in Table 4. The curve shape of each group of mixed powder under each grinding method was similar. The higher the peak height of the overall map, the higher the protein and gluten contents were [24]. With an increase in the amount of tartary buckwheat leaf powder, the peak height of the four doughs showed a downward trend, indicating that the protein content in the dough gradually decreased. Therefore, with the increase in the proportion of tartary buckwheat leaf powder, the role of gliadin and glutelin in the dough was reduced, and the dough had less gluten and elasticity.

With the addition of tartary buckwheat leaf powder, the water absorption of the dough gradually increased (Table 4), which may be due to an increase in dietary fiber content in the leaf powder, resulting in an increase in its water absorption capacity [25]. After adding EMP and UMP to wheat flour, the C2, C3, C4, and C5 values of the dough were lower than those of the control group. With an increased amount of tartary buckwheat leaf powder, the protein weakening rate α increased, the starch gelatinization β decreased, and the enzyme degradation rate γ increased. It can be seen from Table 4 that the protein activity and stability of the dough were affected by the amount of tartary buckwheat leaf powder added and the way of milling, and the ability to change the structure and properties of starch granules was weakened [26,27]. Compared with the amylase hydrolysis rate of the control group, the amylase hydrolysis rate increased with each added amount of EMP, which may be the result of the combined action of the protease contained in the tartary buckwheat leaf powder and the protease in the wheat flour. C3~C4 represented the viscosity attenuation value, and its value was affected by the addition amount of tartary buckwheat leaf powder and the different grinding methods. The overall upward trend indicated that the thermal gelatinization stability of the dough was reduced. With the increase in the leaf powder addition, the C5~C4 value decreased and the retrogradation value increased, which could prolong the shelf life. Under the combined action of mechanical force and temperature, the weakening degree (C2) of the dough protein first decreased and then increased, and the protein weakening rate α gradually decreased with the addition of UMP. Thus, unlike EMP, the addition of UMP reduces the degree of weakening of the dough protein.

After adding SMP or GMP to wheat flour, the trend of the parameters related to dough starch properties was the same as that for EMP and UMP. The retrogradation values of the four methods increased, which was conducive to extending the shelf life of related products. The C2 value of the dough increased first and then decreased with the addition of SMP, but both values were lower than those of the control group, and the protein weakening rate α and the enzyme degradation rate γ showed an overall upward trend. The C2 value of GMP dough showed an upward trend and was higher than that of the control group. The trends observed with α and γ were the same; with an increasing amount of SMP or GMP, the values decreased first and then increased, indicating that the addition of these two kinds of leaf powder could increase the weakening degree of protein in the dough.

**Figure 4:**
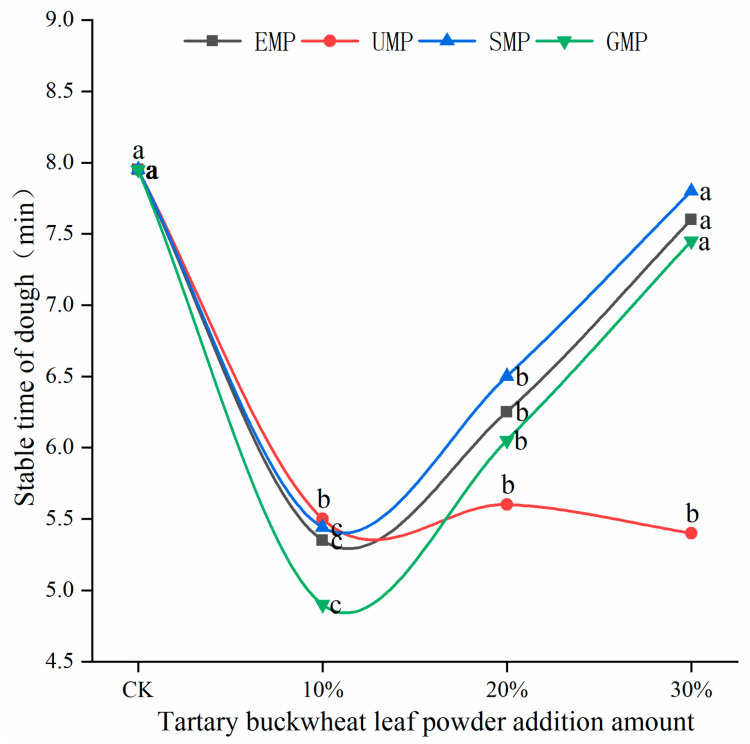
Dough stability times of flour mixed with different amounts of tartary buckwheat powder. Values on the x-axis represent percentages of tartary buckwheat leaf powder in noodles, CK indicates that the addition amount is 0%. Values with different superscript lowercase letters in the same line differ significantly (*p* < 0.05).

The dough stability time of flour mixed with different amounts and milling types of tartary buckwheat leaf powder is shown in Figure 4. The stability time of the dough was affected by the addition amount of tartary buckwheat leaf powder and the different milling methods and the interaction. When the added amount of leaf powder was 10%, the stability time of the dough decreased significantly, and the stability time of the UMP dough was greater than that of the other three leaf powder doughs. The degree of protein weakening was the smallest with UMP, which could make the dough more resistant to kneading, and the extent of damage to the protein structure was the lowest. As the addition of EMP, SMP, and GMP increased from 10% to 30%, the stability time of the corresponding dough gradually increased. However, the addition of UMP over this range of percentages tended to produce a dough with a consistent stability time, which may be due to the fact that some active substances in the ultra-fine tartary buckwheat leaf powder can alter the development of the dough network structure [28].

#### 3.1.6. Effect of Tartary Buckwheat Leaf Powder on Dough Hydration Characteristics

**Figure 5:**
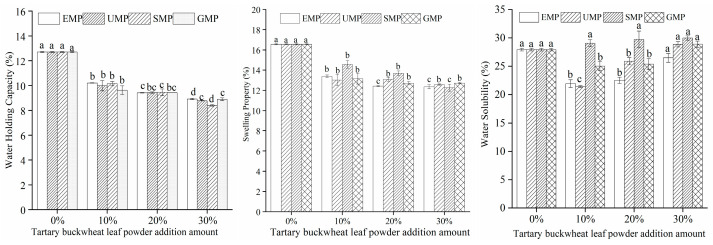
Effect of tartary buckwheat leaf powder addition on dough hydration characteristics. Values with different superscript lowercase letters in the same line differ significantly (*p* < 0.05).

The dough hydration characteristics of different milling methods and different added amounts of tartary buckwheat leaf powder are shown in Figure 5. Under high-temperature conditions, the addition amount of tartary buckwheat leaf powder had a significant effect on the water-holding capacity; when the amount of added leaf powder was 10%, the water-holding capacity of the dough decreased significantly and then gradually decreased with an increasing amount of leaf powder. The addition of tartary buckwheat leaf powder could lead to a decrease in protein content in the dough, thereby causing a decline in its water-holding capacity. The swelling capacity of the dough showed the same trend as the water-holding capacity. The water solubility of the dough was affected by the addition amount of tartary buckwheat leaf powder, the different milling methods, and their interaction. The water solubility of the dough increased with an increase in SMP percentage, while the water solubility trend of the dough was different with EMP, UMP, and GMP. Specifically, when the amount of EMP, UMP, or GMP was 10%, the water solubility of the dough decreased and then gradually increased with an increase in the percentage of powder. Compared to the 0% addition level, the increasing trend in water solubility with the increasing amount of tartary buckwheat leaf powder may be attributed to the interaction between the water-soluble components in the powder and water in the dough, resulting in their dissolution and thereby enhancing the water solubility of the dough. The water solubility of the dough was the lowest with 10% UMP, and the upward trend in water solubility with higher percentages was significantly greater than that with the other two powders. It is indicated that the noodles with 10% UMP may not be mixed with soup seriously.

### 3.2. Effect of Tartary Buckwheat Leaf Powder on Dough Protein Characteristics

#### 3.2.1. Effect of Tartary Buckwheat Leaf Powder on Disulfide Bond and Sulfhydryl Content

**Figure 6:**
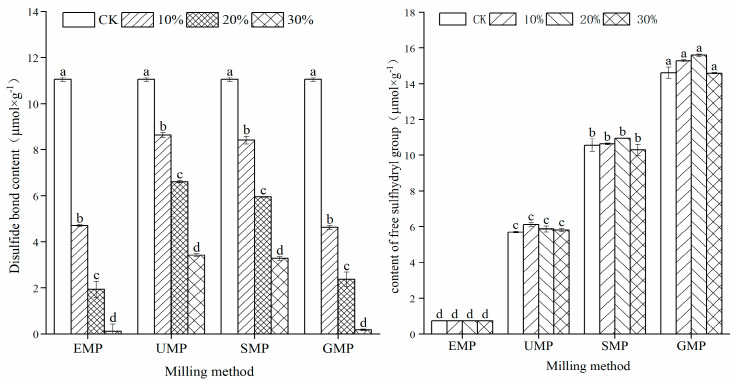
Effect of tartary buckwheat leaf powder addition on disulfide bond and free sulfhydryl content. Values with different superscript lowercase letters in the same line differ significantly (*p* < 0.05).

The sulfhydryl content in different samples is shown in Figure 6. The contents of free sulfhydryl and disulfide bonds were affected by the addition amount of tartary buckwheat leaf powder, the grinding method, and the interaction of the two. Compared with the control group, the addition of four types of tartary buckwheat leaf powder significantly increased the content of free sulfhydryl groups in the samples, which increased with an increase in leaf powder content. When the added amount reached 30%, the free sulfhydryl group content was the highest. In addition, compared with the control group, the disulfide bond content decreased significantly with increased leaf powder percentage. The correlation between free sulfhydryl groups and disulfide bond content in the dough with powder prepared by different milling methods was analyzed. The results showed that the Pearson correlation coefficient between the two was −0.689 with R = 0.013 (*p* < 0.05). This change in the sulfhydryl/disulfide ratio is related to the addition of unique phenolic substances in the tartary buckwheat leaf powder, which reduce the disulfide bonds in the dough, resulting in more free sulfhydryl groups [29]. Thus, the dough network structure was destroyed, and the gluten strength of the dough was lessened. Comparing the four milling methods, the content of disulfide bonds in the dough made by adding UMP was higher than that in the dough made by adding the other three powders. The disulfide bond content is closely related to and is one of the most important factors in maintaining the structure of the gluten protein [30], which is beneficial to dough stability.

#### 3.2.2. Effect of Tartary Buckwheat Leaf Powder on FTIR Spectroscopy

**Figure 7:**
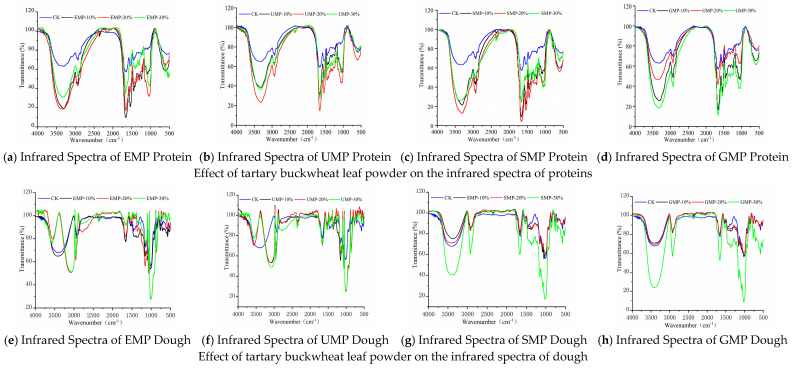
Infrared spectra of proteins and dough from tartary buckwheat leaf powder. (**a**) Infrared spectra of EMP protein; (**b**) infrared spectra of UMP protein; (**c**) infrared spectra of SMP protein; (**d**) infrared spectra of GMP protein; (**e**) infrared spectra of EMP dough; (**f**) infrared spectra of UMP dough; (**g**) infrared spectra of SMP dough; (**h**) infrared spectra of GMP dough. CK indicates that the addition amount is 0%.

The structures of the molecules in the dough were identified by FTIR spectroscopy. The experimental results are shown in Figure 7. Several characteristic peaks were observed as follows. The absorption band at 3500–3200 cm^−1^ (3414 cm^−1^) represents the intermolecular hydrogen bond’s –OH and N–H stretching vibration [31,32]. The absorption peak at 2951 cm^−1^ is the stretching vibration of methine, C–H [33]. The absorption peak at 1663 cm^−1^ belongs to the stretching vibration of C=O and bending vibration of N–H, representing the characteristic absorption of an amide [21]. The absorption peaks at 1372 cm^−1^ and 1245 cm^−1^ are the stretching vibration of C–N and the bending vibration of C–H. The absorption peaks at 1250–1120 cm^−1^ are the stretching vibrations of C–O and C–N and the bending vibration of C–O–C. The absorption peaks at 860 cm^−1^ and 765 cm^−1^ are the bending vibrations of N–H [34].

It can be seen from Figure 7e–g and h that the infrared spectra of the dough were significantly different when the tartary buckwheat leaf powder prepared by different milling methods was added to the wheat flour at different percentages, especially when EMP and UMP were added. In addition to the common characteristic peaks, when 10%, 20%, and 30% UMP (Figure 7f) were added to the dough, the single peak at 3414 cm^−1^ representing the –OH and N–H stretching vibration changed into two peaks at 3568 cm^−1^ and 3100 cm^−1^, which were attributed to the intramolecular association of hydroxyl groups; when 20% and 30% EMP were used (Figure 7e), new absorption peaks also appeared. There are three possible reasons for these observations. First, the addition of leaf powder enhanced the intermolecular hydrogen bonding force after mixing stone milled leaf powder and wheat flour, and Fermi resonance occurred, causing the spectrum to split, resulting in two peaks in the infrared spectrum [35]. Second, the two substances combined well after the addition of stone milled leaf powder, resulting in a new absorption peak. From the results of scanning electron microscopy (Figure 11a), it can also be seen that when 20% EMP was added, the two substances were closely associated. Third, components in the added leaf powder cross-linked with components in the wheat flour to produce new functional groups. However, when considering Figure 7a–d), there was no significant change in the characteristic peaks of the corresponding proteins in the four groups of infrared spectra, excluding the possibility of the creation of new functional groups in the proteins of wheat flour.

After adding different amounts of SMP and GMP to the dough, except for the common characteristic peaks, the infrared spectra were roughly the same, indicating that the addition of these two leaf powders did not produce new functional groups and did not change the internal structures.

#### 3.2.3. Effect of Tartary Buckwheat Leaf Powder on Protein Secondary Structure

According to the method of Chen et al. [12], the amide I band (1600–1700 cm^−1^) obtained from each infrared spectrum was subjected to baseline correction, correction, and deconvolution using OMNIC 8.0 software and then fitted with a Gaussian second derivative using PeakFit 4.0 software until the iteration value was 7, R^2^ > 0.99. According to the peak attribution, the relative area of each secondary structure was calculated. Specifically, 1650–1658 cm^−1^ is an α-helix, 1600–1640 cm^−1^ is a β-sheet, 1660–1700 cm^−1^ is a β-turn, and 1640–1650 cm^−1^ is a random coil. The α-helix structure and β-sheet structure are considered to be the most stable secondary structures to form an ordered network structure [36,37].

**Figure 8:**
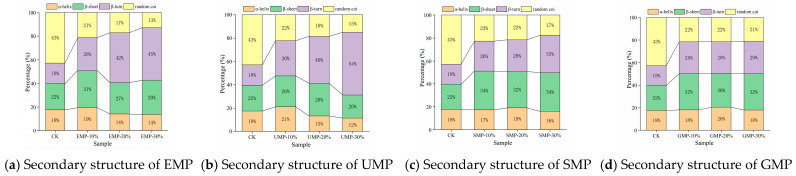
Effect of tartary buckwheat leaf powder on secondary structure changes. (**a**) Secondary structure of EMP; (**b**) secondary structure of UMP; (**c**) secondary structure of SMP; (**d**) secondary structure of GMP. CK indicates that the addition amount is 0%.

The four structural contents were affected by the amount of tartary buckwheat leaf powder added, the milling method, and the interaction. From Figure 8, it can be seen that the α-helix content decreased as the added amount of EMP or UMP increased. The addition of increasing amounts of tartary buckwheat leaf powder from the other two grinding methods led to an increase and then a decrease in the α-helix structure of the dough. The content of the α-helical structure was the highest with 10% UMP, indicating that there were more ordered structures in the dough. The trend in β-sheet change was different with UMP than with the tartary buckwheat leaf powder prepared by the other three grinding methods. Specifically, the β-sheet structure increased first and then decreased when the added amount of UMP reached 20%. Under the four milling methods, the relative content of β-turn and random coil structures showed an opposite trend overall. The relative content of β-turn structures showed an upward trend, while that of random coil structures showed a downward trend. The changes in secondary structures may be related to the competition for water between gluten and fiber [37].

#### 3.2.4. Results of Two-Way ANOVA

It can be seen from the Table 5 that the grinding method has no significant effect on the water-holding force, C5–C4, the protein weakening rate α, and has a very significant effect on all other indexes. The amount of bitter buckwheat leaf powder had a significant effect on all indices. However, the interaction between the grinding mode and the amount of tartary buckwheat leaf powder added also had no significant effect on water retention, C5–C4, and protein weakening rate α.

#### 3.2.5. Effect of Tartary Buckwheat Leaf Powder on Fluorescence Spectra

**Figure 9:**
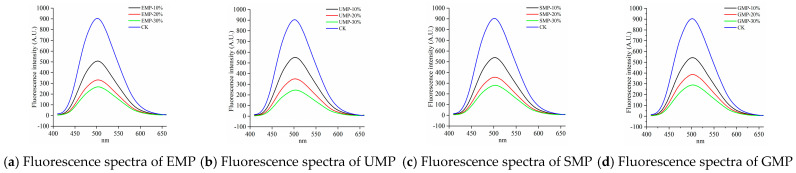
Fluorescence spectra of dough with different added amounts of tartary buckwheat leaf powder. (**a**) Fluorescence spectra of EMP; (**b**) fluorescence spectra of UMP; (**c**) fluorescence spectra of SMP; (**d**) fluorescence spectra of GMP. CK indicates that the addition amount is 0%.

As shown in Figure 9, compared with the control, the fluorescence emission peak intensity of the samples after adding tartary buckwheat leaf powder was significantly reduced and gradually decreased with increasing amounts of added leaf powder. This indicates that the addition of tartary buckwheat leaf powder reduced the surface hydrophobic area in the dough and changed the conformation of the dough protein. Other possibilities are that the hydrophobic groups in the dough protein aggregated and the low-molecular-weight proteins polymerized to form a macromolecular polymer, or the addition of tartary buckwheat leaf powder destroyed disulfide bonds, exposing hydrophobic amino acid residues in the dough that cross-linked with other active ingredients in the leaf powder [36]. Therefore, the addition of tartary buckwheat leaf powder could lead to the destruction of disulfide bonds in the dough; the dough protein could then combine with the proteins and cellulose in the leaf powder to form macromolecular aggregates, inhibiting the formation of network structures, and thereby reducing the strength of the dough protein.

**Figure 10:**
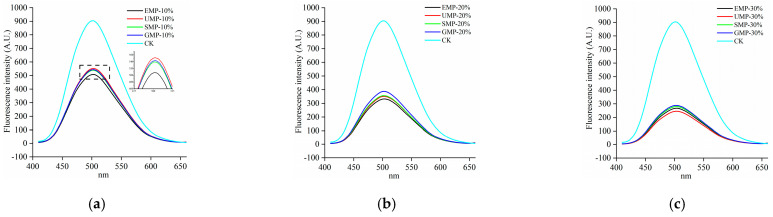
Fluorescence spectra of dough under different milling methods. (**a**) Fluorescence spectra of 10% tartary buckwheat powders; (**b**) fluorescence spectra of 20% tartary buckwheat powders; (**c**) fluorescence spectra of 30% tartary buckwheat powders. CK indicates that the addition amount is 0%.

The fluorescence spectra of the dough by grinding method are shown in Figure 10. At 10% added tartary buckwheat leaf powder, the hydrophobic regions were slightly higher with the ultra-fine grinding than with the other three grinding methods. A possible reason for this observation is that the content of disulfide bonds in UMP was high and fewer low-molecular-weight proteins aggregated to form new polymers.

#### 3.2.6. Effect of Tartary Buckwheat Leaf Powder on SEM Images

**Figure 11:**
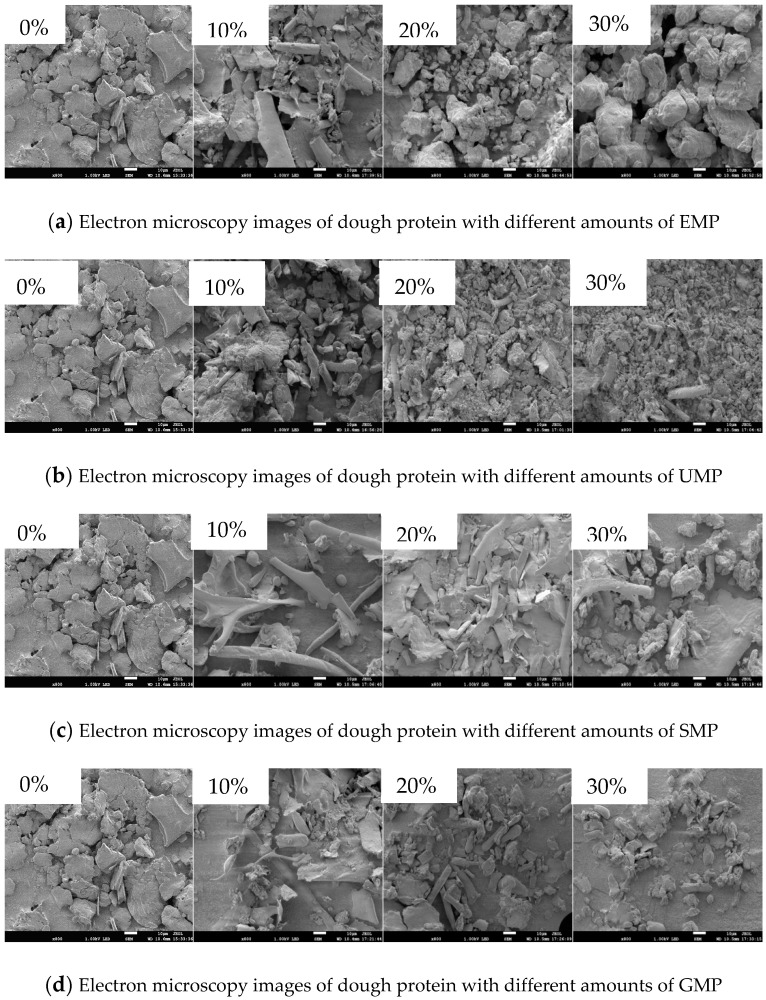
Effect of tartary buckwheat leaf powder on scanning electron microscope images. (**a**) Electron microscopy images of dough protein with different amounts of EMP; (**b**) electron microscopy images of dough protein with different amounts of UMP; (**c**) electron microscopy images of dough protein with different amounts of SMP; (**d**) electron microscopy images of dough protein with different amounts of GMP (scanning electron micrographs of dough protein at 800×).

The effects of tartary buckwheat leaf powder on the SEM images of dough protein are shown in Figure 11. At 10% EMP (Figure 11a), most of the protein structures existed in irregular flakes and were unevenly distributed. At 20% EMP, the granular structure increased, the surface was rough, and there were numerous holes. At 30% EMP, the block-like irregular structures became larger and the surface was relatively smooth, with small particles embedded in it. In addition, there were numerous holes of uniform size. The results showed that the 10% EMP tartary buckwheat leaf powder was not closely associated with the wheat flour, and most of the powder existed separately. However, with an increase in the added amount, the association of the tartary buckwheat leaf powder and wheat flour improved, which increased the number of block-like particles corresponding to new functional groups in the 20% EMP infrared spectrum. The increased number of holes may be due to the addition of leaf powder resulting in less protein content in the dough, diluting the dough protein and hindering the formation of disulfide bonds.

At 10% UMP (Figure 11b), there were numerous rod-like particles accompanied by unevenly distributed particles with block-like morphologies. At 20% UMP, there were small granules and rods, and the distribution was more homogenous. At 30% UMP, the protein particles were reduced, the overall structure was dense and uniform, and there were individual rod-like structures. This indicates that ultra-fine milling of the leaf powder can make it associate with the wheat flour, changing the protein characteristics, and thus altering the dough quality.

At 10% SMP and GMP (Figure 11c and Figure 11d, respectively), the protein structures were unevenly distributed and dense, and small particles were attached to thinner rod-like structures. At 20% SMP and GMP, the rod-like structures increased and existed as a thin film, but they were more evenly distributed. At 30% SMP and GMP, there were irregular particles, large pores, and voids on the granular surface, all with an uneven distribution. This shows that the steel mill and grain mill leaf flour could not closely associate with the wheat flour. When the added amount reached 30%, the protein structure changed, showing a particularly poor structural form.

Therefore, the addition of 10% UMP produced a different structure from that of the other three leaf powders, and 20% EMP also changed the structure of the dough protein.

## 4. Conclusions

The addition of tartary buckwheat leaf powder had a significant effect on the physicochemical properties and structure of dough protein. With an increase in tartary buckwheat leaf powder, the hydration characteristics, protein weakening rate, and starch gelatinization characteristics of the dough changed, and the water-holding capacity and swelling capacity decreased. The retrogradation value increased, which could prolong the shelf life of related products. The 10% UMP dough is the least water soluble, and 10% UMP noodles are not prone to soup mixing; the degree of protein denaturation is the smallest, with the highest content of disulfide bonds, and the infrared spectrum changes from a single peak to a double peak, there is a higher content of the α-helical structure, and the hydrophobic regions were slightly higher than other proportions. Considering the effects of different milling methods, different added amounts of tartary buckwheat leaf powder, and their interaction effects on various characteristics of dough, 10% UMP is the most suitable amount to add to the dough. After that, we can further investigate 10% UMP noodles and continue exploring the effects of adding 10% UMP on noodle quality characteristics.

## Figures and Tables

**Table 1:** Basic components of tartary buckwheat leaf powder obtained by different milling methods.

Basal Component	Milling Method			
	EMP	UMP	SMP	GMP
Ash content %	11.37 ± 0.03 ^b^	11.05 ± 0.01 ^c^	10.60 ± 0.05 ^d^	11.79 ± 0.04 ^a^
Protein %	23.00 ± 0.27 ^bc^	24.11 ± 0.31 ^a^	22.37 ± 0.24 ^c^	23.53 ± 0.25 ^ab^
Soluble dietary fiber %	6.06 ± 0.03 ^c^	6.95 ± 0.07 ^a^	5.39 ± 0.09 ^d^	6.29 ± 0.02 ^b^
Insoluble dietary fiber %	22.44 ± 0.07 ^b^	21.58 ± 0.10 ^d^	22.84 ± 0.04 ^a^	22.17 ± 0.02 ^c^
Rutin mg·100 g^−1^	4310 ± 0.57 ^c^	4853 ± 0.59 ^a^	4489 ± 0.67 ^b^	4333 ± 0.51 ^bc^
Quercetin mg·100 g^−1^	110 ± 0.00 ^b^	160 ± 0.00 ^a^	110 ± 0.00 ^b^	120 ± 0.00 ^b^
γ-aminobutyric acid mg·100 g^−1^	112 ± 0.87 ^bc^	119 ± 1.17 ^a^	107 ± 3.09 ^b^	114 ± 0.18 ^b^
Chlorophyll mg·100 g^−1^	454 ± 0.00 ^bc^	540 ± 0.09 ^a^	469 ± 0.00 ^b^	443 ± 0.09 ^c^

Data represent the mean of three independent experiments ± standard deviation (*n* = 3). Values with different superscript lowercase letters in the same line differ significantly (*p* < 0.05). Electric stone mill powder (EMP), ultra-fine mill powder (UMP), steel mill powder (SMP), and grain mill powder (GMP).

**Table 2:** Particle size and specific surface area of tartary buckwheat leaf powder by different milling methods.

Milling Method	D50/μm	Specific Surface Area/m^2^·kg^−1^	Cell Disruption Rate/%
EMP	2.59 ± 0.05 ^d^	1057.7 ± 18.50 ^a^	386.8 ± 1.64 ^a^
UMP	3.27 ± 0.03 ^c^	868.9 ± 8.20 ^b^	305.7 ± 0.59 ^b^
SMP	4.37 ± 0.02 ^b^	683.8 ± 1.79 ^c^	296.7 ± 0.09 ^c^
GMP	13.65 ± 0.06 ^a^	496.2 ± 11.53 ^d^	73.6 ± 0.03 ^d^

Data represent the mean of three independent experiments ± standard deviation (*n* = 3). Values with different superscript lowercase letters in the same column differ significantly (*p* < 0.05).

**Table 3:** Hydration characteristics of tartary buckwheat leaf powder obtained with different milling methods.

Milling Method	Water-Holding Capability g·g^−1^		Water Solubility %		Swelling Property g·g^−1^	
	25 °C	100 °C	25 °C	100 °C	25 °C	100 °C
EMP	5.28 ± 0.01 ^b^	6.81 ± 0.05 ^b^	20.42 ± 0.05 ^a^	30.95 ± 0.24 ^b^	3.25 ± 0.00 ^d^	3.06 ± 0.00 ^c^
UMP	4.45 ± 0.05 ^c^	4.78 ± 0.00 ^c^	19.82 ± 0.04 ^c^	29.37 ± 0.14 ^c^	3.43 ± 0.00 ^c^	3.12 ± 0.00 ^d^
SMP	4.24 ± 0.26 ^c^	4.86 ± 0.29 ^c^	20.10 ± 0.03 ^b^	32.27 ± 0.20 ^a^	4.08 ± 0.00 ^b^	4.64 ± 0.00 ^b^
GMP	6.65 ± 0.07 ^a^	9.87 ± 0.65 ^a^	18.06 ± 0.08 ^d^	24.43 ± 0.20 ^d^	5.23 ± 0.00 ^a^	7.15 ± 0.00 ^a^

Data represent the mean of three independent experiments ± standard deviation (*n* = 3). Values with different superscript lowercase letters in the same column differ significantly (*p* < 0.05).

**Table 4:** Rheological parameters of flour mixed with tartary buckwheat leaf powder at different percentages.

Sample	Water Absorption	Stable Time of Dough/min	C2/Nm	C3/Nm	C4/Nm	C5/Nm	C3–C4/ Nm	C5–C4/ Nm	α/ (Nm/min)	β/ (Nm/min)	γ/ (Nm/min)
CK	60% ± 0.00 ^d^	7.95 ± 0.35 ^a^	0.59 ± 0.00 ^a^	2.03 ± 0.01 ^a^	1.93 ± 0.05 ^a^	3.26 ± 0.00 ^a^	0.10 ^m^	1.33 ^a^	−0.06 ± 0.00 ^a^	0.56 ± 0.01 ^a^	−0.02 ± 0.00 ^ab^
EMP-10%	70% ± 0.00 ^c^	5.35 ± 0.07 ^de^	0.45 ± 0.01 ^ef^	1.93 ± 0.03 ^b^	1.71 ± 0.03 ^b^	2.29 ± 0.07 ^b^	0.22 ^k^	0.58 ^b^	−0.06 ± 0.01 ^a^	0.55 ± 0.01 ^b^	−0.03 ± 0.04 ^bc^
EMP-20%	76% ± 0.00 ^b^	6.25 ± 0.07 ^b^	0.45 ± 0.03 ^ef^	1.81 ± 0.05 ^f^	1.36 ± 0.04 ^f^	1.78 ± 0.06 ^e^	0.45 ^e^	0.42 ^c^	−0.07 ± 0.01 ^a^	0.35 ± 0.02 ^i^	−0.05 ± 0.01 ^de^
EMP-30%	83% ± 0.00 ^a^	7.60 ± 0.00 ^a^	0.48 ± 0.00 ^d^	1.67 ± 0.00 ^i^	1.09 ± 0.00 ^i^	1.40 ± 0.00 ^gh^	0.58 ^a^	0.31 ^e^	−0.07 ± 0.00 ^a^	0.31 ± 0.00 ^k^	−0.07 ± 0.00 ^fg^
UMP-10%	70% ± 0.00 ^c^	5.50 ± 0.28 ^cd^	0.43 ± 0.00 ^g^	1.92 ± 0.01 ^b^	1.67 ± 0.00 ^c^	2.25 ± 0.03 ^c^	0.25 ^j^	0.58 ^b^	−0.07 ± 0.00 ^a^	0.50 ± 0.04 ^e^	−0.03 ± 0.02 ^abc^
UMP-20%	78% ± 0.00 ^b^	5.55 ± 0.35 ^cd^	0.41 ± 0.00 ^h^	1.75 ± 0.00 ^g^	1.31 ± 0.00 ^h^	1.72 ± 0.00 ^f^	0.44 ^f^	0.41 ^c^	−0.06 ± 0.00 ^a^	0.40 ± 0.04 ^h^	−0.05 ± 0.01 ^def^
UMP-30%	84% ± 0.00 ^a^	5.35 ± 0.21 ^de^	0.44 ± 0.00 ^f^	1.58 ± 0.01 ^k^	1.04 ± 0.00 ^k^	1.34 ± 0.01 ^i^	0.54 ^b^	0.30 ^e^	−0.06 ± 0.00 ^a^	0.31 ± 0.00 ^k^	−0.08 ± 0.01 ^g^
SMP-10%	70% ± 0.00 ^c^	5.44 ± 0.23 ^de^	0.44 ± 0.00 ^f^	1.90 ± 0.00 ^c^	1.64 ± 0.00 ^d^	2.29 ± 0.00 ^b^	0.26 ^i^	0.65 ^b^	−0.06 ± 0.00 ^a^	0.54 ± 0.00 ^c^	−0.01 ± 0.00 ^a^
SMP-20%	75% ± 0.00 ^b^	6.50 ± 0.00 ^b^	0.50 ± 0.00 ^c^	1.83 ± 0.01 ^e^	1.42 ± 0.00 ^e^	1.86 ± 0.01 ^d^	0.41 ^g^	0.44 ^c^	−0.07 ± 0.01 ^a^	0.46 ± 0.00 ^f^	−0.03 ± 0.00 ^bc^
SMP-30%	84% ± 0.00 ^a^	7.80 ± 0.14 ^a^	0.50 ± 0.00 ^c^	1.59 ± 0.00 ^j^	1.07 ± 0.01 ^j^	1.39 ± 0.02 ^h^	0.52 ^c^	0.32 ^de^	−0.08 ± 0.01 ^a^	0.33 ± 0.03 ^j^	−0.06 ± 0.01 ^efg^
GMP-10%	70% ± 0.00 ^c^	4.90 ± 0.00 ^e^	0.48 ± 0.00 ^d^	1.87 ± 0.00 ^d^	1.66 ± 0.02 ^c^	2.28 ± 0.01 ^b^	0.21 ^l^	0.62 ^b^	−0.06 ± 0.00 ^a^	0.52 ± 0.01 ^d^	-0.04 ± 0.00 ^cd^
GMP-20%	78% ± 0.00 ^b^	6.05 ± 0.07 ^bc^	0.46 ± 0.01 ^e^	1.71 ± 0.02 ^h^	1.34 ± 0.02 ^g^	1.74 ± 0.00 ^f^	0.37 ^h^	0.40 ^cd^	−0.06 ± 0.01 ^a^	0.43 ± 0.05 ^g^	−0.03 ± 0.01 ^bc^
GMP-30%	84% ± 0.00 ^a^	7.45 ± 0.21 ^a^	0.53 ± 0.00 ^b^	1.60 ± 0.00 ^j^	1.10 ± 0.00 ^i^	1.41 ± 0.00 ^g^	0.50 ^d^	0.31 ^e^	−0.07 ± 0.00 ^a^	0.31 ± 0.02 ^k^	−0.07 ± 0.01 ^g^

Data represent the mean of three independent experiments ± standard deviation (*n* = 3). Values with different superscript lowercase letters in the same column differ significantly (*p* < 0.05).

**Table 5:** The interaction effect of milling method and addition amount on the characteristic value.

Mill Method	Significance	Add the % of Tartary Buckwheat Leaf Powder	Significance	Mill Method * Add the % of Tartary Buckwheat Leaf Powder	Significance
Content of free sulfhydryl group	**	Content of free sulfhydryl group	**	Content of free sulfhydryl group	**
Disulfide bond content	**	Disulfide bond content	**	Disulfide bond content	**
Stable time of dough	**	Stable time of dough	**	Stable time of dough	**
Water Holding Capacity	ns	Water-Holding Capacity	**	Water-Holding Capacity	ns
Water Solubility	**	Water Solubility	**	Water Solubility	**
Swelling Property	*	Swelling Property	**	Swelling Property	*
C2	**	C2	**	C2	**
C3	**	C3	**	C3	**
C4	**	C4	**	C4	**
C5	**	C5	**	C5	**
C3–C4	**	C3–C4	**	C3–C4	**
C5–C4	ns	C5–C4	**	C5–C4	ns
α	ns	α	*	α	ns
β	**	β	**	β	**
γ	**	γ	**	γ	**
α-helix	**	α-helix	**	α-helix	**
β-sheet	**	β-sheet	**	β-sheet	**
β-turn	**	β-turn	**	β-turn	**
random coil	**	random coil	**	random coil	**

** indicates extremely significant difference (*p* < 0.01), * indicates significant effect (*p* < 0.05), and ns indicates no significant difference.

## Data Availability

The original contributions presented in the study are included in the article, further inquiries can be directed to the corresponding author.

## References

[1] Bao G., Zhao Y., Zheng Q., Zhou H. (2015). Microwave digestion-inductively coupled plasma atomic emission spectrometry simultaneous determination of 13 elements in tartary buckwheat leaves. Chin. Inorg. Anal. Chem..

[2] Krzysztof D., Danuta G., Artur S., Hanna S., Ivan K., Elżbieta G., Jarosław W. (2018). The Content of Dietary Fibre and Polyphenols in Morphological Parts of Buckwheat (*Fagopyrum tataricum*). Plant Foods Hum. Nutr..

[3] Tuan P.A., Thwe A.A., Kim J.K., Kim Y.B., Lee S., Park S.U. (2013). Molecular characterisation and the light-dark regulation of carotenoid biosynthesis in sprouts of tartary buckwheat (*Fagopyrum tataricum Gaertn*.). Food Chem..

[4] Cheng F., Ge X., Gao C., Li Y., Wang M. (2019). The distribution of D-chiro-inositol in buckwheat and its antioxidative effect in HepG2. J. Cereal Sci..

[5] Li H. (2010). Study on Antioxidant Function and Utilization of Tartary Buckwheat Powder and Leaf Powder. Master’s Thesis.

[6] Liu E., Liang L., Zhang S. (2001). Production of baked health foods from plant leaves containing flavonoids. Food Ind..

[7] Ren Y., Yao B., Guo Y., Yang Z., Wei D., Xu X. (2017). Research and process optimization of tartary buckwheat leaf lotus leaf detoxification health meal replacement powder. Agric. Prod. Process..

[8] Zhang Y., Zhang M., Guo X., Bai X., Zhang J., Huo R., Zhang Y. (2023). Improving the adsorption characteristics and antioxidant activity of oat bran by superfine grinding. Food Sci. Nutr..

[9] Liu J., Luo D., Li X., Xu B., Zhang X., Liu J. (2016). Effects of inulin on the structure and emulsifying properties of protein components in dough. Food Chem..

[10] Chen S., Ni Z., Thakur K., Wang S., Zhang J., Shang Y., Wei Z. (2021). Effect of grape seed power on the structural and physicochemical properties of wheat gluten in noodle preparation system. Food Chem..

[11] Han C., Ma M., Zhang H., Li M., Sun Q. (2020). Progressive study of the effect of superfine green tea, soluble tea, and tea polyphenols on the physico-chemical and structural properties of wheat gluten in noodle system. Food Chem..

[12] Sun J., Chen M., Hou X., Li T., Qian H., Zhang H., Li Y., Qi X., Wang L. (2021). Effect of phosphate salts on the gluten network structure and quality of wheat noodles. Food Chem..

[13] Tao B., Ye F., Li H., Hu Q., Xue S., Zhao G. (2014). Phenolic Profile and In Vitro Antioxidant Capacity of Insoluble Dietary Fiber Powders from Citrus (*Citrus junos Sieb. ex Tanaka*) Pomace as Affected by Ultrafine Grinding. J. Agric. Food Chem..

[14] Zhao X., Du F., Zhu Q., Qiu D., Yin W., Ao Q. (2010). Effect of superfine pulverization on properties of *Astragalus membranaceus* powder. Powder Technol..

[15] Zhong C., Zu Y., Zhao X., Li Y., Ge Y., Wu W., Zhang Y., Li Y., Guo D. (2016). Effect of superfine grinding on physicochemical and antioxidant properties of pomegranate peel. Int. J. Food Sci. Technol..

[16] Zhu F., Du B., Xu B. (2015). Superfine grinding improves functional properties and antioxidant capacities of bran dietary fibre from Qingke (hull-less barley) grown in Qinghai-Tibet Plateau, China. J. Cereal Sci..

[17] Zhao X., Liu H., Zhang X., Ao Q. (2018). Effect of pressure grinding technology on the physicochemical and antioxidant properties of *Tremella aurantialba* powder. J. Food Process Pres..

[18] Reddy D.H.K., Harinath Y., Seshaiah K., Reddy A.V.R. (2010). Biosorption of Pb(II) from aqueous solutions using chemically modified Moringa oleifera tree leaves. Chem. Eng. J..

[19] Stani C., Vaccari L., Mitri E., Birarda G. (2020). FTIR investigation of the secondary structure of type I collagen: New insight into the amide III band. Spectrochim. Acta Part A Mol. Biomol. Spectrosc..

[20] Zhao X., Zhu H., Chen J., Ao Q. FTIR, XRD and SEM Analysis of Ginger Powders with Different Size. J. Food Process Pres..

[21] Zhao X., Meng A., Zhang X., Liu H., Guo D., Zhu Y. (2020). Effects of ultrafine grinding on physicochemical, functional and surface properties of ginger stem powders. J. Sci. Food Agric..

[22] Cao Y., Zhang M., Dong S., Guo P., Li H. (2020). Impact of potato pulp on the processing characteristics and gluten structures of wheat flour dough. J. Food Process Pres..

[23] Arif S., Ahmed M., Chaudhry Q., Hasnain A. (2018). Effects of water extractable and unextractable pentosans on dough and bread properties of hard wheat cultivars. LWT.

[24] Yang L., Zhang H., Huang B., Hao S., Li S., Li P., Yu H. (2023). Studying the Role of Potato Powder on the Physicochemical Properties and Dough Characteristics of Wheat Flour. Gels.

[25] Ding J., Hou G.G., Nemzer B., Xiong S., Dubat A., Feng H. (2018). Effects of controlled germination on selected physicochemical and functional properties of whole-wheat flour and enhanced γ-aminobutyric acid accumulation by ultrasonication. Food Chem..

[26] Noort M., Van H., Hemery Y., Schols H., Hamer R. (2010). The effect of particle size of wheat bran fractions on bread quality—Evidence for fibre–protein interactions. J. Cereal Sci..

[27] Girard A., Bean S., Tilley M., Adrianos S., Awika J. (2018). Interaction mechanisms of condensed tannins (*Proanthocyanidins*) with wheat gluten proteins. Food Chem..

[28] Zhao L. (2012). Study on the Effect of Frozen Storage on Molecular Weight, Chain Structure and Aggregation State of Gluten Protein. Ph.D. Thesis.

[29] Wang P., Chen H., Mohanad B., Xu L., Ning Y., Xu J., Wu F., Yang N., Jin Z., Xu X. (2014). Effect of frozen storage on physico-chemistry of wheat gluten proteins: Studies on gluten-, glutenin- and gliadin-rich fractions. Food Hydrocoll..

[30] Niu H., Zhang M., Xia X., Liu Q., Kong B. (2018). Effect of porcine plasma protein hydrolysates on long-term retrogradation of corn starch. Food Chem..

[31] Diop C., Li H., Xie B., Shi J. (2011). Impact of the catalytic activity of iodine on the granule morphology, crystalline structure, thermal properties and water solubility of acetylated corn (*Zea mays*) starch synthesized under microwave assistance. Ind. Crop Prod..

[32] Li G., Gao X., Wang Y., He S., Guo W., Huang J. (2023). Effects of superfine grinding sweet potato leaf powders on physicochemical and structure properties of sweet potato starch noodles. Food Sci. Nutr..

[33] Bryukvina L., Ivanov N. (2016). Fermi resonance of molecular complexes with strong hydrogen bond in irradiated LiF:OH crystals. J. Fluor. Chem..

[34] Chen G., Ehmke L., Sharma C., Miller R., Faa P., Smith G., Li Y. (2019). Physicochemical properties and gluten structures of hard wheat flour doughs as affected by salt. Food Chem..

[35] Liu L., Shi Z., Wang X., Ren T., Ma Z., Li X., Xu B., Hu X. (2021). Interpreting the correlation between repeated sheeting process and wheat noodle qualities: From water molecules movement perspective. LWT.

[36] Guo X., Sun X., Zhang Y., Wang R., Yan X. (2018). Interactions between soy protein hydrolyzates and wheat proteins in noodle making dough. Food Chem..

[37] Zhou Y., Dhital S., Zhao C., Ye F., Chen J., Zhao G. (2021). Dietary fiber-gluten protein interaction in wheat flour dough: Analysis, consequences and proposed mechanisms. Food Hydrocoll..

